# Exploration of consumer preference based on deep learning neural network model in the immersive marketing environment

**DOI:** 10.1371/journal.pone.0268007

**Published:** 2022-05-04

**Authors:** Qiang Zheng, Qingshan Ding

**Affiliations:** 1 School of Economics and Management, Ningxia University, Yinchuan, China; 2 Business School, University of Huddersfield, Huddersfield, United Kingdom; Hanyang University, REPUBLIC OF KOREA

## Abstract

The study intends to increase the marketing quantity of various commodities and promote the comprehensive development of the market. The study first discusses the principle and current situation of the emerging Immersive Marketing. Then, it analyzes the Deep Learning (DL) Neural Network (NN) model. Finally, a Personalized Recommendation System (PRS) is designed based on the Immersive Marketing environment using the Graph Neural Network (GNN) model. The proposed PRS based on the Immersive Graph Neural Network (IGNN) model has reflected higher advantages over other recommendation systems. The experiment results suggest that Immersive Marketing can fully reflect commodities’ essential attributes and characteristics, improve users’ shopping experience, and promote sales. Meanwhile, the IGNN-based PRS reported here gives users an elevated and immersive shopping experience and entertainment process. Lastly, the model comparison finds that the proposed IGNN outperforms other models. The optimal model parameters are verified as P@20 and R@20 to gain the highest composite index values. In particular, parameter R@20 gives the model a better performance over P@20. The study provides technical references for improving the marketing process of various commodities and entertainment products and contributes to marketing technology development.

## Introduction

With the development of the times and science and technological progress, commodity marketing is experiencing dramatic changes, and users’ shopping experience is also constantly improving. Inspired by science and technology, an immersive marketing environment has gradually entered daily shopping activities. An Immersive Marketing environment can profoundly reflect the characteristics and attributes of goods. It can also comprehensively improve users’ shopping experience to increase sales [1]. In particular, Deep Learning (DL) Neural Network (NN) model is introduced into Personalized Recommendation System (PRM) for an Immersive Marketing environment. The Immersive Marketing environment can comprehensively improve users’ shopping experience and promote commodity marketing [2, 3]. Although the practical application in this field has not been popularized, many studies have provided technical support.

Li et al. (2022) proposed a novel Particle Swarm Optimization (PSO) algorithm for dynamically adjusting controller parameters. By dynamically changing the learning factor, the particles carefully searched their neighborhood in the early stage of the algorithm to prevent them from missing the global optimum and converging to the local optimum. In the later stage of evolution, the particles would converge to the fast and accurate global optimum solution to accelerate the convergence. Finally, four different controllers under different navigation conditions were simulated through the experiment. The results showed that the controller based on the algorithm had the advantages of slight overshoot, short adjustment time, accurate control, and strong interference immunity [4]. Ran et al. (2021) developed a new K-Means Clustering (KMC) algorithm based on noise to capture urban hotspots. Using noise algorithm, adding noise judgment, they randomly enhanced the attributes of data points and clustering outputs. As a result, the number of clusters and the initial central cluster were automatically obtained. The results of four unsupervised cluster analyses were used to verify the results. The Nonparametric Wilcoxon statistical analysis method was used to verify the distribution state and difference in clustering results. Finally, five taxi real-time location data sets from Aracaju (Brazil), San Francisco (United States), Rome (Italy), Chongqing (China), and Beijing (China) were selected to test and verify the effectiveness of the proposed KMC noise algorithm. Then, the algorithm was compared with fuzzy C-means, K-means, and K-means plus methods. The comparative experiments found that the KMC noise algorithm could reasonably obtain the cluster numbers and the initial central cluster. The proposed KMC noise algorithm demonstrated excellent clustering performance, accurately obtained the clustering results and effectively captured urban hot spots [5]. Cui et al. (2021) devised a new fault diagnosis method based on Variational Mode Decomposition (VMD) and Maximum Correlation Kurtosis Deconvolution (MCKD) for the rolling body of VMD rolling bearing: VMD-MCKD. The vibration signal of the rolling bearing was decomposed into natural mode functions by the VMD method. Then, the Kurtosis criterion determined the number of modes with prominent faults to calculate the deconvolution period. The sensitivity MCKD method enhanced the periodic fault element of the reconstructed signal. Finally, the power spectrum of the reconstructed signal was analyzed in detail to obtain the fault frequency and diagnose the rolling element faults. The simulation signal and actual vibration signal were selected to verify the effectiveness of the reported VMD-MCKD method. The experiment revealed that VMD-MCKD could effectively diagnose the rolling element faults with high accuracy [6].

Based on the above literature review, this paper first deeply discusses Immersive Marketing. Then, the DL model is discussed, and the PRS for Immersive Marketing based on Graph Neural Network (GNN) model is designed. Finally, the estimation results of the model are studied and compared. The innovation is to design an Immersive Marketing model based on the DL model, which comprehensively creates a new way to apply science and technology. This study provides technical references for improving the current marketing environment and contributes to market development.

## Research theories and methods

### Immersive marketing

With the deepening economic globalization, Chinese enterprises face the dual development pressure of domestic and international competition. In order to catch up with market development and improve operating efficiency, enterprises must pay more attention to marketing. "Immersiveness" refers to an immersive feeling that allows people to focus on their current concerns [7]. Immersive Experience combines the qualitative and quantitative aspects and interprets users’ psychophysiological needs in a digital space. The aim is to reconstruct users’ interactive experiences in hearing, touch, vision, smell, force, and motion perception. At the same time, it can also meet users’ needs for a universal and barrier-free experience and combine high-tech, multimedia, and sensor technology. The Immersive Experience lets users engage in a virtual space where they can perform actions like manipulating, choosing, and modifying. This real-time perception effect offers users a convenient design and Experience, bringing higher consumer satisfaction [8]. Furthermore, in an Immersive Experience or an intoxicating experience, users concentrate on a specific activity while ignoring external interference and reaching a state of absolute engagement. Such an experience method can bring users a sense of enrichment and happiness. Therefore, this experience method is also defined as a deep-level User Experience [9].

**Fig 1:**
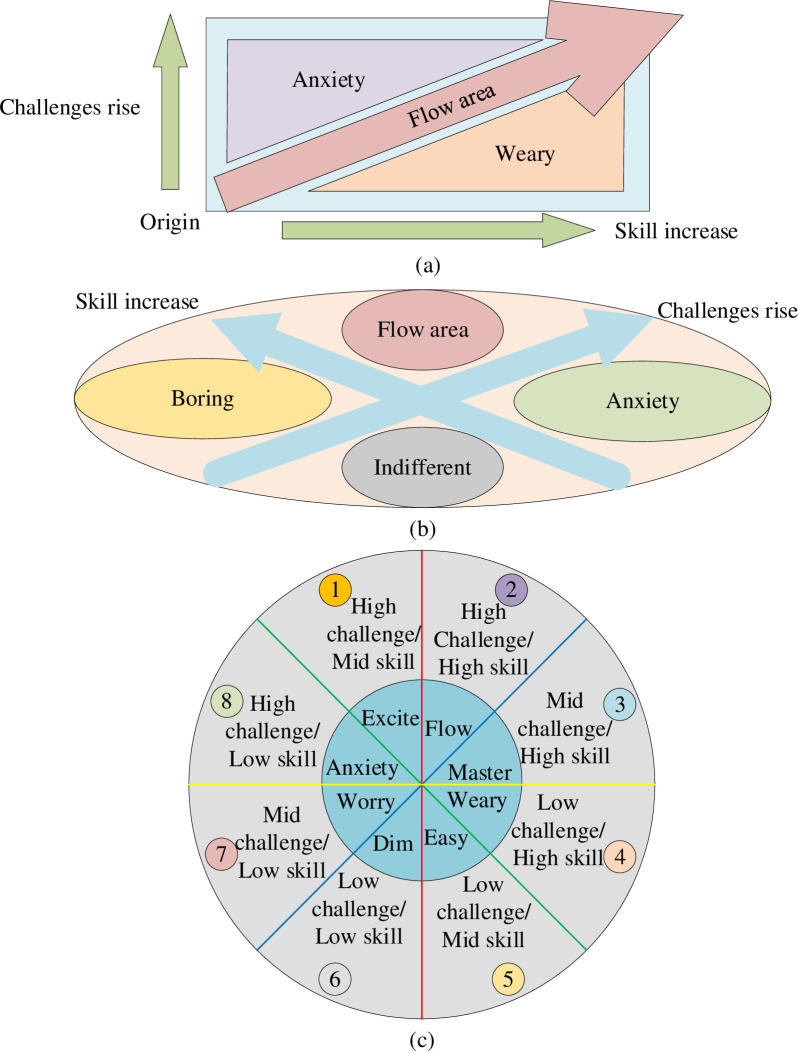
Flow Theory framework of Immersive Experience (a: three-channel, b: four-channel, c: eight-channel).

The research on Immersive Experience often varies according to specific application fields. In marketing, Immersive Experience is usually used for immersive shopping and entertainment. Immersive Experience based on Virtual Reality (VR) technology can elevate User Experience and reconstruct an Immersive Marketing environment. In particular, the complexity of social behavior, interactive behavior, and user perception will affect user Immersive Experience [10]. Meanwhile, Immersive Marketing relies on a virtual digital space to minimize the natural world’s impact on users’ Immersive Experience. The core theory of Immersive Experience is the Flow Theory: the positive psychological Experience produced by a specific activity [11]. Psychological Flow Theory has three-channel, four-channel, and eight-channel models, as shown in Fig 1.

As in Fig 1, the human Experience is intuitively described in different channels. Importantly, the description of flow state (Experience) varies according to different models. Immersive Marketing can recommend products to users in real-time by evaluating users’ psychological states. It drives User Experience toward Immersive Experience to improve the marketing effect [12]. The three-channel model has a relatively simple structure and describes user flow Experience using general psychology. Thus, it is difficult to capture the user’s actual psychological flow pattern [13]. By comparison, the eight-channel psychological flow model extends the three-channel model to better capture user psychological flow [14]. Meanwhile, the eight-channel flow model describes human psychological states more in detail and involves broader and more specific psychology. Thus, the eight-channel flow model can accurately and effectively reflect and capture the user’s psychological flow Experience [15].

### Deep Learning (DL) Neural Network (NN) model

DL is a Deep Neural Network (DNN) structure that learns and analyzes data by simulating the working mechanism of the human brain. DL can complete the data calculation tasks. Essentially, DL methods and technologies extract features from complex data, streamline data analysis and comprehensively analyze the overall data features through centralized analysis. Thereby, they complete data processing by constructing multi-level and nonlinear change models [16]. A deep network structure is a technology for data interpretation and analysis. Its main task is to complete feature learning. Differently put, it learns various data processing methods through data accumulation. It continuously optimizes the system through accumulation and error statistics. Finally, DL realizes the highly accurate and intelligent Machine Learning (ML) [17]. The learning mechanism of DL is mainly divided into two parts. The first step is unsupervised learning from top to bottom. The algorithm starts from the bottom and trains parameters at all levels with unlabelled data, unlike the supervised algorithm for training Shallow Neural Network (SNN). Generally, the DNN model is a three-layer system. The following is a whole DL NN training process. Firstly, the six-dimensional vector is imported. Two hidden layers output a two-dimensional vector: the probability that the input samples belong to one of the two categories. Before unsupervised learning, the system initializes the network parameters randomly. It optimizes the parameters through layer-wise training to get them close to data features. Eventually, the optimal NN parameters are obtained. After the first layer’s optimal parameters, the feature learning can continue upward and only retains the first and second hidden layers of the original NN. An output layer is added after the second hidden layer. Based on this, the eigenvalues learned by the first layer are fed to the second layer, obtaining the training parameters with minimum model errors. In this way, the second-layer feature expression of input data is obtained, which signifies an end of a training cycle. Nevertheless, the DNN model training in practical application is more complex for a more accurate feature expression and prediction. Compared with shallow ML, DL has more advantages. First, the DL model has more hidden layers and nodes. A deeper model structure often means higher computation complexity and data-processing power. Hence, DL improves ML’s speed and data processing efficiency [18]. Secondly, the DL model highlights autonomous feature learning in task processing rather than manual feature extraction. Deep features can more accurately reflect the internal relationship of data [19].

**Fig 2:**
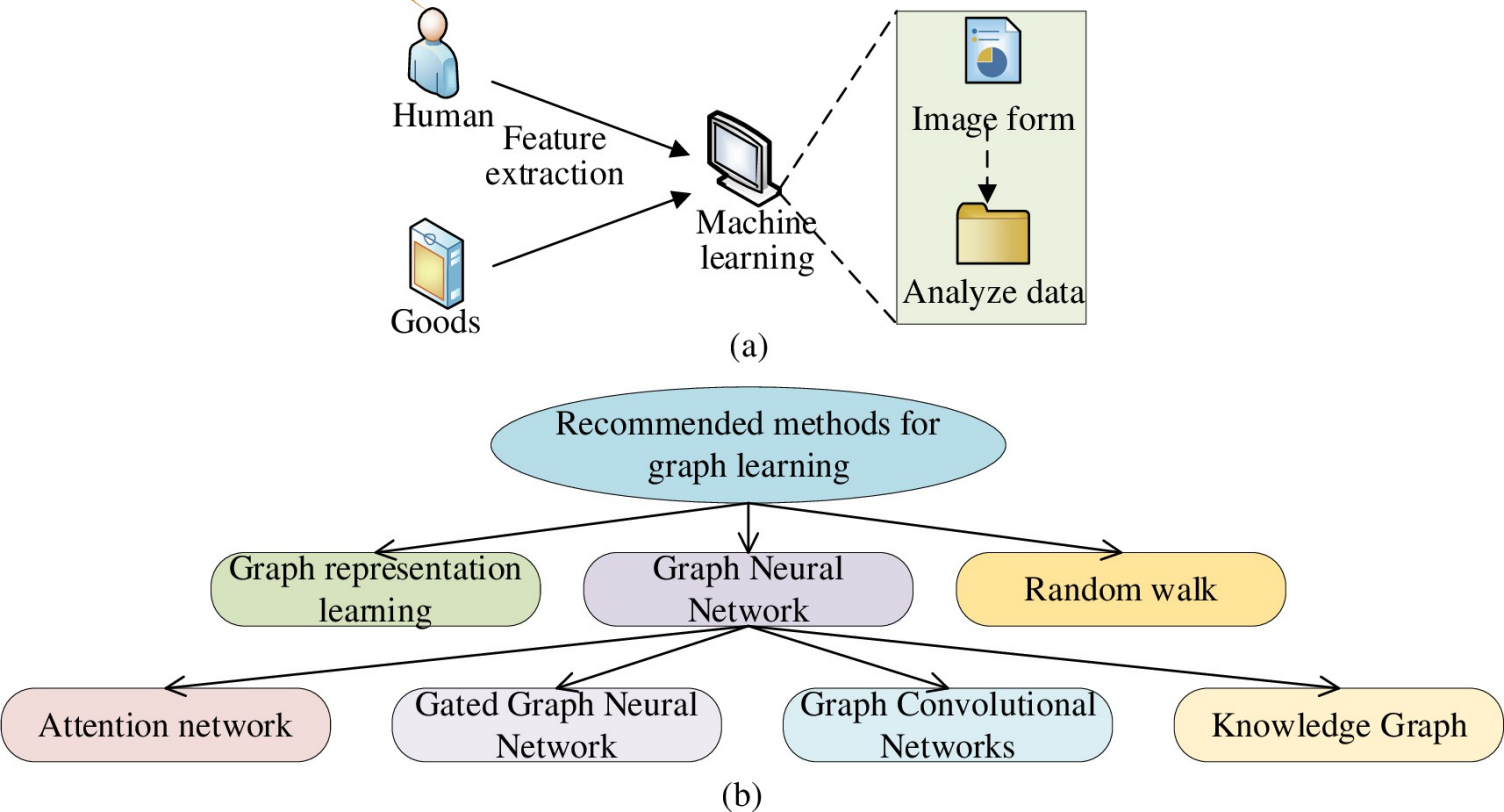
Basic principle and classification of GL-based PRS (a is the basic principle, b is the classification).

As an advanced AI technology in DL, Graph Learning (GL) method has developed rapidly in recent years and envisions a good application prospect. Its primary connotation is the graph structure-specific ML method. The application of the GL method in PRS is one direction in GL development because most data in PRS have a graph structure. For example, the user’s evaluation of items and the attribute information between users is a bipartite graph [20]. Therefore, it is necessary to consider the complexity of objects in designing PRS, and the GL method has great advantages in learning and handling complex object relationships. In particular, Graph Neural Network (GNN) is widely used in PRS’s graph structure data analysis [21]. Fig 2 details the principle and classification of GL-based PRS.

As in Fig 2, the GL method has a wide application and diverse categories. GNN is an efficient representation learning framework based on the graph data structure. GNN follows the rule of neighbor information aggregation; the target and its adjacent nodes’ features are aggregated, represented by a feature vector. GNN mainly has three operations: Aggregate, Combine, and Readout [22]. Through the graph structure analysis, a GNN with a k-layer can be defined as:

$$a_{v}^{\left(k\right)}=AGGREGATE^{\left(k\right)}\left(\left\{h_{u}^{\left(k-1\right)}:u\in N\left(v\right)\right\}\right)$$ (1)

$$h_{v}^{\left(k\right)}=COMBINE^{\left(k\right)}\left(h_{v}^{\left(k-1\right)},a_{v}^{\left(k\right)}\right),h_{v}^{\left(0\right)}=X_{v}$$ (2)

In Eqs (1) and (2), $h_{v}^{\left(k\right)}$ represents the vector representation of node **v** after **k**-times feature processing. **N(v)** refers to the aggregation vector representation of node **v** neighbor nodes, and **u** denotes other nodes of the neural network structure. Moreover, the specific contents of the two operations **AGGREGATE**^(**k**)^ and **COMBINE**^(**k**)^ are very important for the node representation in the GNN structure. Eq (3) defines **AGGREGATE**^(**k**)^ in the GNN structure:

$$a_{v}^{\left(k\right)}=MAX\left(\left\{ReLU\left(W\cdot h_{u}^{\left(k-1\right)}\right),\forall u\in N\left(v\right)\right\}\right)$$ (3)

In Eq (3), **W** represents the learned parameter matrix, and **MAX** is the maximum node region. **AGGREGATE** and **OMBINE** operations can be summarized as follows:

$$h_{v}^{\left(k\right)}=ReLU\left(W\cdot MEAN\left\{h_{u}^{\left(k-1\right)},\forall u\in N\left(v\right)\cup \left\{v\right\}\right\}\right)$$ (4)

In Eq (4), the meanings of all parameters are consistent with those in the above equation. The multilayer network propagation rule of Graph Convolution Neural Network (GCNN) is expressed as [23]:

$$H^{\left(1+1\right)}=\sigma \left({\tilde{D}}^{-\frac{1}{2}}\tilde{A}{\tilde{D}}^{-\frac{1}{2}}H^{\left(1\right)}W^{\left(1\right)}\right)$$ (5)

$$\tilde{A}=A+I_{N}$$ (6)

**Fig 3:**
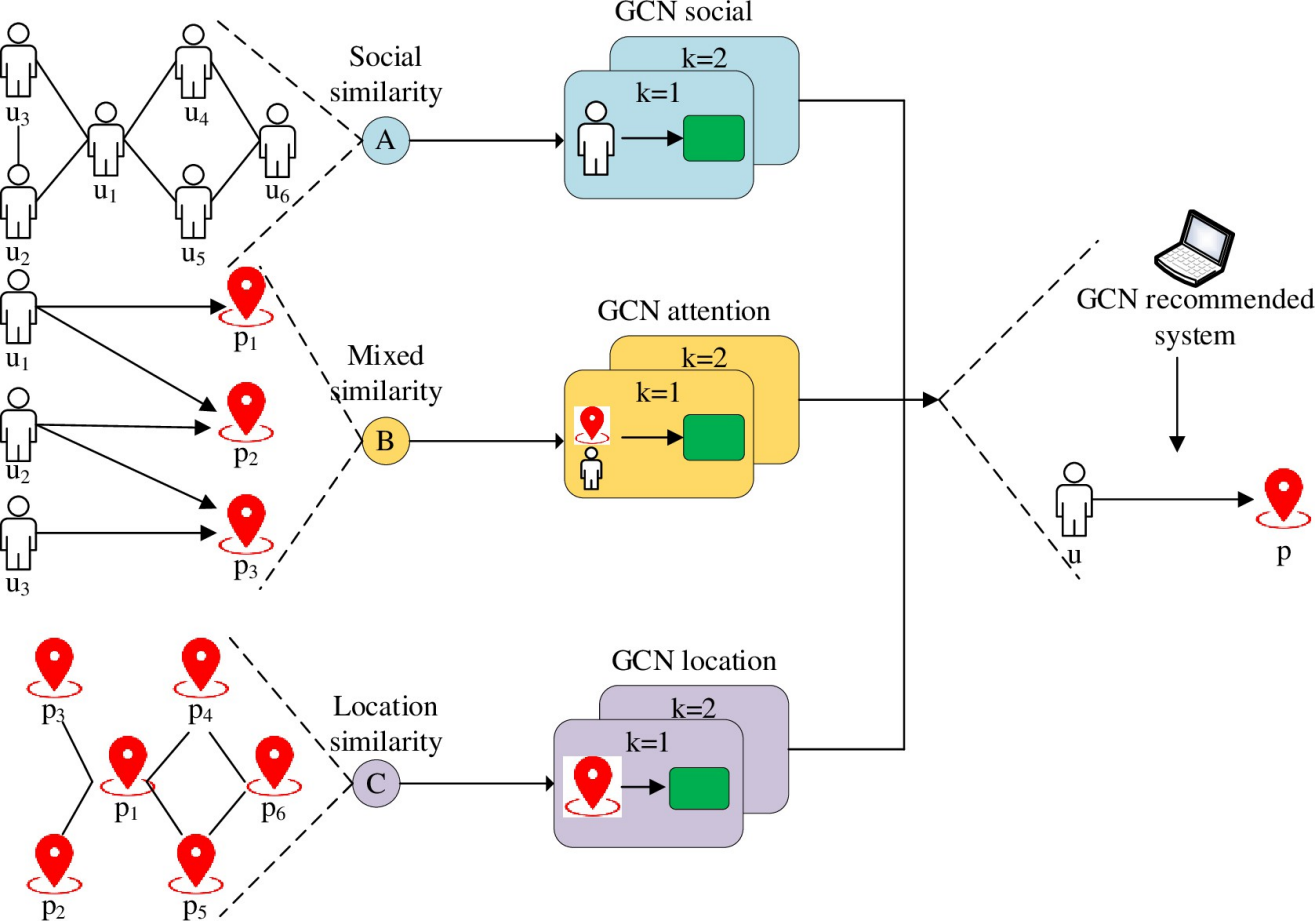
GCNN-based feature extraction framework.

Eq (6) represents the self-connected adjacency matrix, ***I***_***N***_ is the identity matrix, and **W**^(**1**)^ indicates the weight matrix. $\tilde{D}$ stands for the Activation Function (AF). By defining and extracting user and commodity features, GCNN recommends users with more suitable and timely commodities. Fig 3 sketches the main flow of user feature extraction by GCNN.

**Fig 4:**
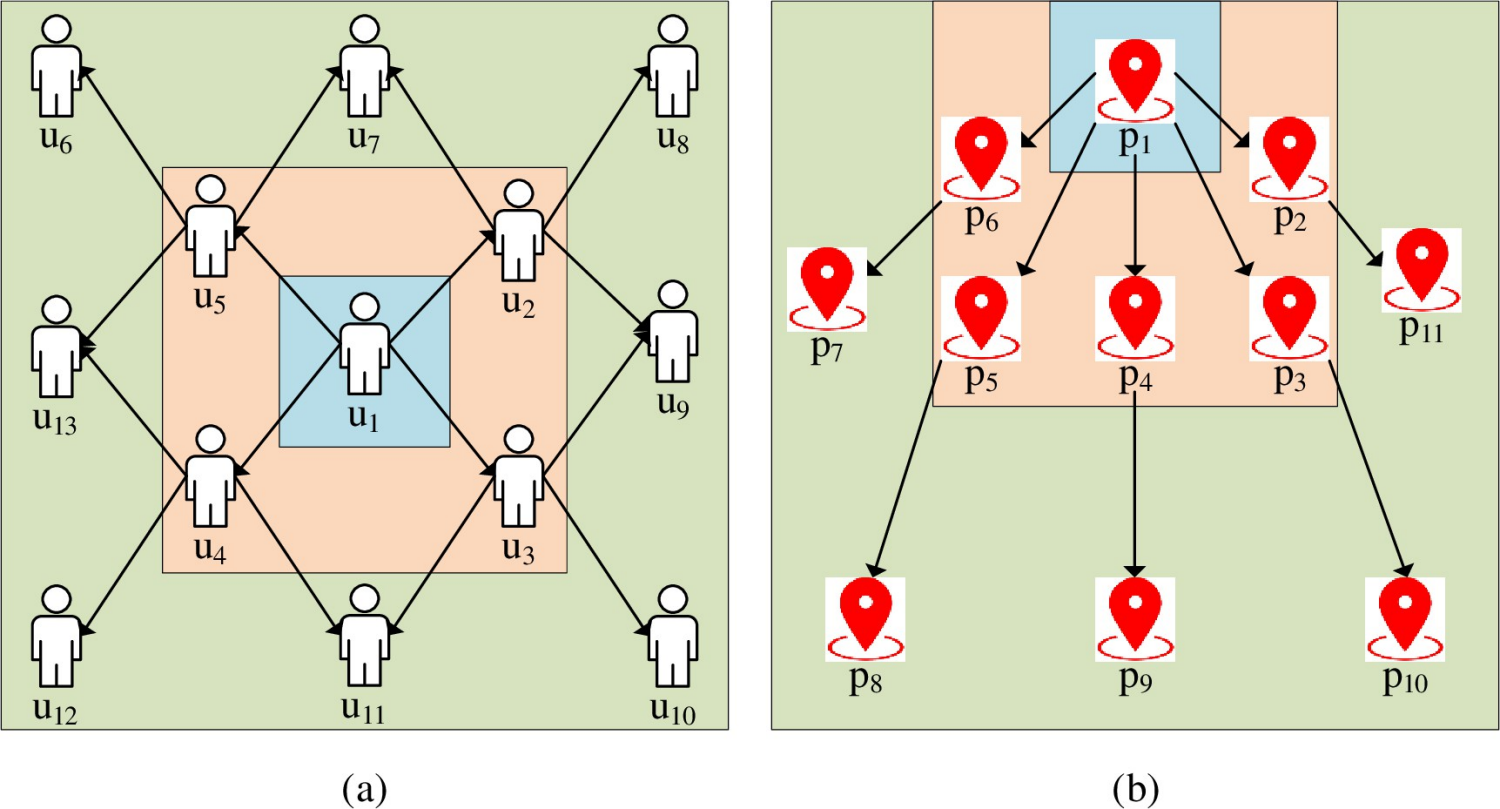
Impact of user POI (a: social impact; b: environmental impact).

From Fig 3, users’ different Points of Interest (POI) are closely related to their social interactions and environment. Comprehensively analyzing users’ POI and social interactions can grasp users’ preferences and help optimize PRS. Then, Fig 4 displays the influence of user POI.

Fig 4 analyzes users’ main POIs from social intercourse and browsing records. Then, analyzing every neighbor comprehensively step-by-step provides a basic reference for the model.

### Graph Convolution Neural Network (GCNN)-based recommendation model

Here, GCNN obtains the user’s POI and analyzes the network spatial structure information. First, the user browsing record is analyzed, calculated by Eq (7) through the network location [24]:

$$a_{i,j}=exp\left(-\eta ∥d_{i}-d_{j}{∥}^{2}\right)$$ (7)

In Eq (7), ***η*** is the geographical relevancy. ***i*** and j are the main longitude and latitude of the user’s location. The greater **a**_***i*,*j***_ is, the closer the two positions are. The calculation of the adjacency matrix reads:

$$S={\tilde{D}}^{\left(-\frac{1}{2}\right)}\tilde{A}{\tilde{D}}^{\left(-\frac{1}{2}\right)}$$ (8)

In Eq (8), $\tilde{D}$ is the degree matrix of $\tilde{A}$, namely, a basic range of $\tilde{A}$. Their meanings are the same as the above equation. Secondly, users’ social relationships and preferences are estimated by modeling users as network nodes. Since the adjacency matrix values in the traditional GCNN are discrete and ∈ {0,1}, the estimated user preference has a large error. Suppose a* _***i*,*j***_ represents the GCNN adjacency matrix. In that case, users’ browsing similarity with their friends must be analyzed based on users’ network locations and circle of friends [25]. When two users are not friends, the environmental similarity between user **u**_*i*_ and **u**_***j***_ is calculated by Eq (9):

$$a^{*}{}_{i,j}=\frac{\left|R_{i}\cap R_{j}\right|}{\left|R_{i}\cup R_{j}\right|}$$ (9)

In Eq (9), ***R*** represents the location set of browsing content. If two users are friends, their social similarity can be calculated by Eq (10):

$$a^{*}{}_{i,j}=\beta \frac{\left|R_{i}\cap R_{j}\right|}{\left|R_{i}\cup R_{j}\right|}+\left(1-\beta \right)\frac{\left|F_{i}\cap F_{j}\right|}{\left|F_{i}\cup F_{j}\right|}$$ (10)

In Eq (10), ***F*** represents the set of the user’s circle of friends. ***β*** ∈ [0,1], which is the user similarity balance parameter. Further, the trained model will be used for prediction and be optimized. First, users’ POI will be predicted. Usually, multiple POIs can be aggregated around a specific POI because of user curiosity. Accordingly, the users’ POI will be analyzed from two aspects: the impact of users’ browsing location on user POIs, and the impact of social relations on users’ POIs [26]. In order to predict user POI from browsing locations, the user POI location is vectorized, as in Eq (11):

$${\hat{j}}_{i}=\left[c_{1}P_{\left(*,1\right)},\ldots ,c_{n}P_{\left(*,n\right)}\right]$$ (11)

In Eq (11), **P**_(*,n)_ represents the element in the **n**th column of the POI matrix, and it is also the position of the **n**th POI. **c** denotes the browsing position POI. The calculation of environmental impact by introducing the user browsing location reads:

$$f_{i}=sum\;\left(Score\cdot \left(j_{i}\cdot W^{\left(4\right)}\right)\right)$$ (12)

In Eq (12), **sum**, **Score**, and **W**^(**4**)^ are the addition function, user POI matrix, and the weight parameter matrix. ***f***_***i***_ means the score vector of user ***u***_***i***_. From the users’ perspective, the scoring vector of user POI is calculated by combining user POI and user social interaction. The specific calculation reads [27]:

$$z_{i}=w_{a}\cdot \;\text{Concat}\;\left(v_{i},u_{i}\right)$$ (13)

$$e_{i}=MLP\;\left(z_{i}\right)$$ (14)

In Eq (14), ***v***_***i***_, ***u***_***i***_, and ***z***_***i***_ represent the user POI, user’s social interaction, and the input. ***MLP*** is the Multilayer Perceptron, namely, the multilayer analysis process of NN structure. Then, a list of POIs is provided for users through analysis and is calculated by Eq (15):

$${\hat{y}}_{i}=sigmoid\left(f_{i}+e_{i}\right)$$ (15)

In Eq (15), **sigmoid** represents the AF. ${\hat{y}}_{i}$ is the predicted score of all users on all POI, and the interest conformity is judged by the score [28].

### Research data

This paper mainly models the interest recommendation node based on GCNN and the user Attention Mechanism (AM). It adopts the Immersive Experience marketing method to calculate the user’s POIs and provide users with items more in line with their POIs to improve the marketing effect. Then, the model is trained by Book-Crossings, Yelp, and Foursquare data sets and is evaluated by Map, Precision, and Recall. Different data sets can evaluate the model performance and comprehensively improve the learning ability of the model. At the same time, this paper also analyzes the influence of parameters through ablation experiments [29]. Table 1 lists the data set information used here.

**Table 1:** Basic information of experimental dataset.

Number	Index	Book-Crossings	Yelp	Foursquare
1	Subscribers	90000	30887	24941
2	Points of Interest	27000	18995	28593
3	Visit	1100000	860888	1196248
4	Density (%)	0.1523	0.1399	0.1006

As in Table 1, the Book-crossings dataset is a Book-scoring dataset compiled by Cai-Nicolas Ziegler according to book information. Yelp dataset is a collection of business-related user comments and user information. The Foursquare dataset contains the recorded data of the places browed by users. The calculation of the three evaluation indexes reads:

$$\text{precision}@,\;k=\frac{1}{m}\sum_{u=1}^{m}\frac{P_{u}\left(k\right)\cap T_{u}}{k}$$ (16)

$$\text{recall}@,\;k=\;\frac{1}{m}\sum_{u=1}^{m}\frac{P_{u}\left(k\right)\cap T_{u}}{\left|T_{u}\right|}$$ (17)

$$\text{map}@,\;k=\frac{1}{m}\sum_{u=1}^{m}\frac{\sum_{j=1}^{k}S\left(j\right)\times f\left(j\right)}{\left|T_{u}\right|}$$ (18)

In Eqs (16–18), **k, S,** and **f** represent the user interest node, sorting accuracy, and the index function, respectively. Meanwhile, (precision@, k) is the proportional of POI under visit by the user. (recall@, k) denotes the proportion of POI visited by the user. (map@, k) indicates the mean Precision. Then, the model reported here is verified by comparing it with Gate Recurrent Unit (GRU) [30], User Preference & Time Sequence-based POI Recommendation (UPTS-Prec) [31], Location-Based Social Network (LBSN), Session-based Recsys-Graph Neural Network (SR-GNN) [32], and Multidimensional Context-Aware Graph Embedding (MCAGE) [33]. The model reported here is the Immersive Graph Neural Network (IGNN).

## Evaluation of deep learning model under immersive marketing

### Immersive marketing environment

**Fig 5:**
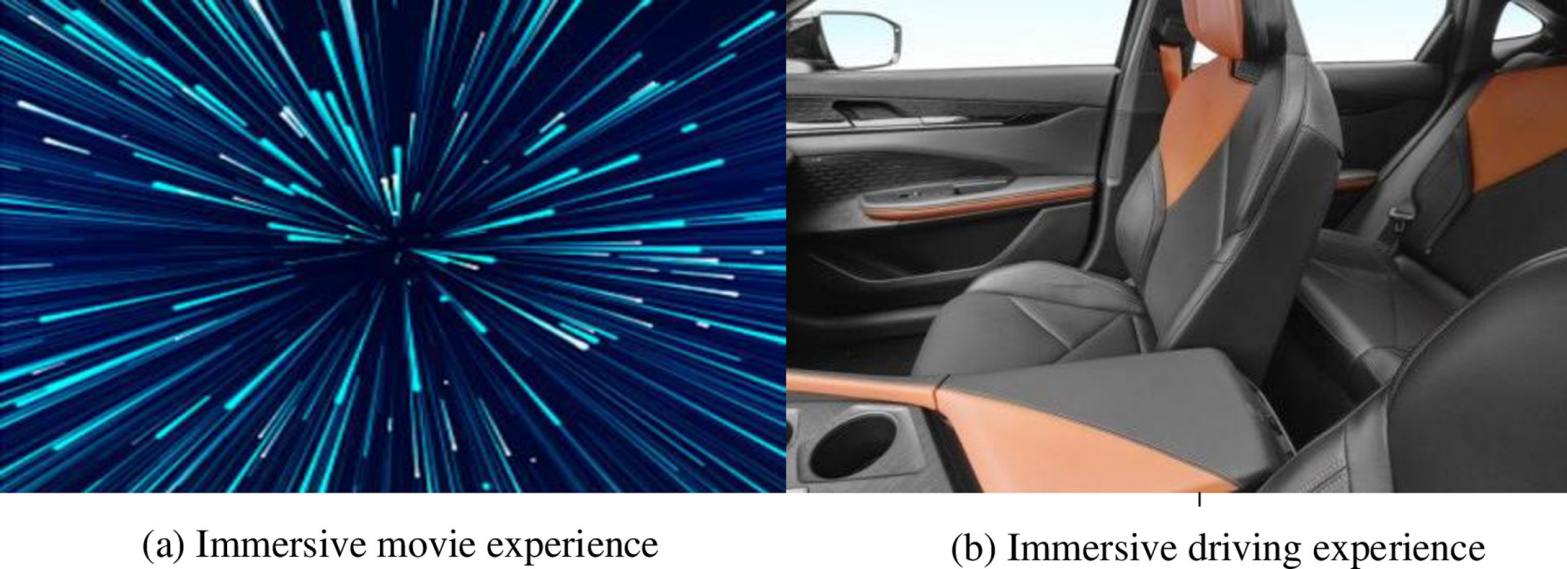
Immersive marketing.

As an advanced marketing method, Immersive Marketing has unique characteristics to attract users, bring an intuitive User Experience, and reflect products’ attributes. While increasing the User Experience, it can also promote marketing. Fig 5 explains the Immersive Marketing process for various products.

As in Fig 5, Immersive Marketing has become the leading marketing method in the business and entertainment industries. Thus, based on the DL recommendation model, strengthening the marketing strength can promote Immersive Marketing more extensively.

### Deep learning recommendation system under immersive marketing

**Fig 6:**
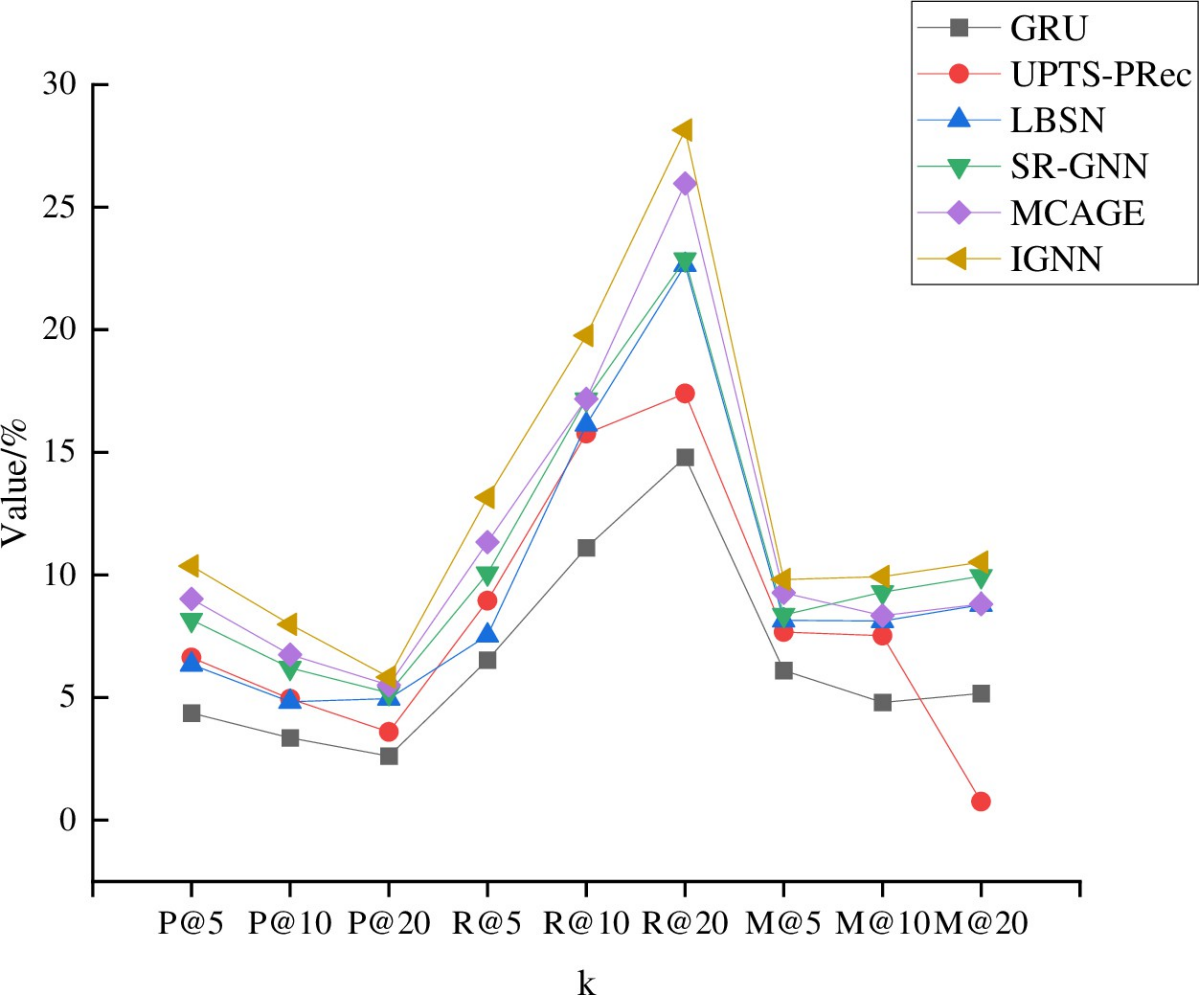
Model training results on Book-Crossings dataset.

Subsequently, the model is trained by the Book-Crossings, Yelp, and Foursquare datasets, setting k as 5, 10, and 20, respectively. Fig 6 describes the model training results on the Book-Crossings dataset.

**Fig 7:**
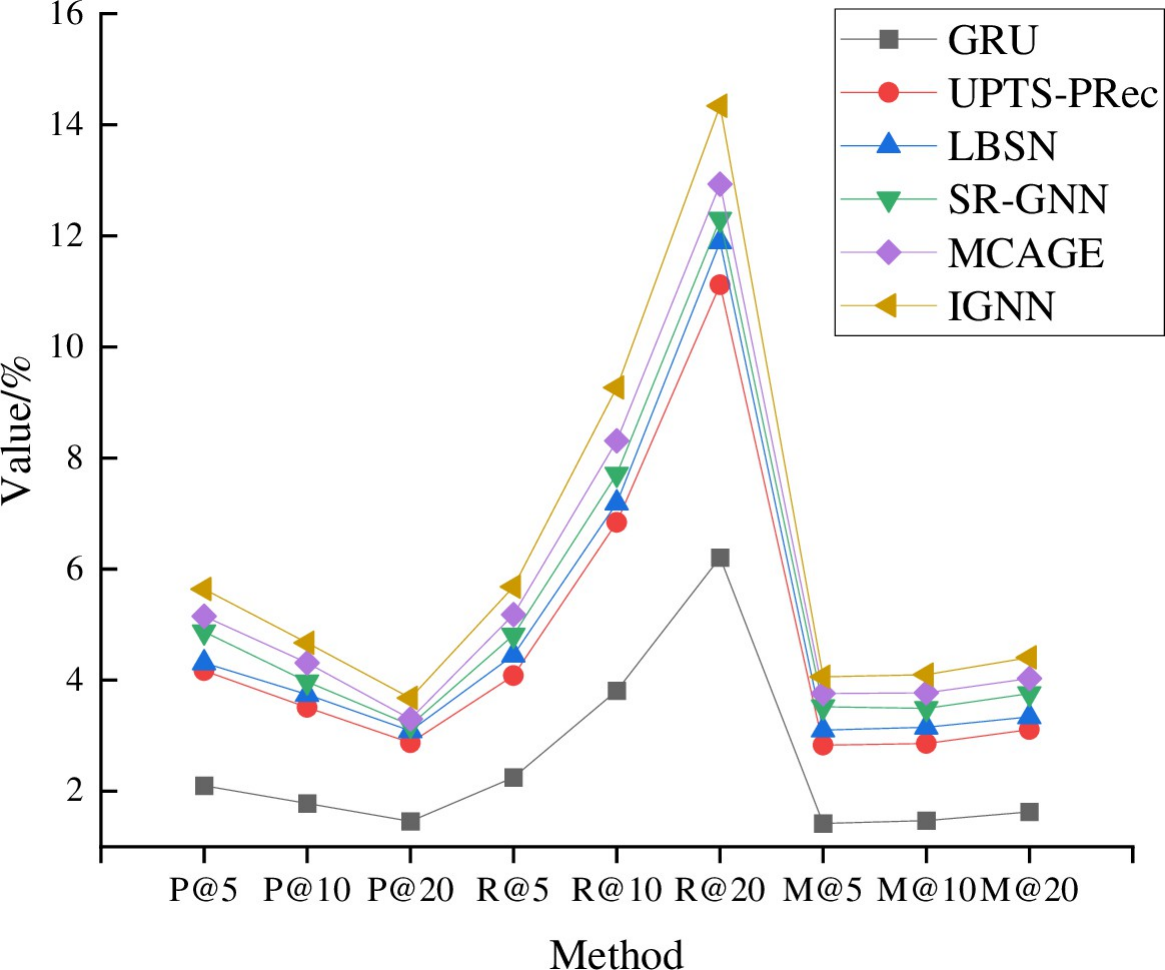
Model training results on Yelp dataset.

Fig 6 compares the training results of each model using the Book-Crossings dataset. P, R, and M represent Precision, Recall, and Map. Apparently, under the parameter conditions of 5, 10, and 20, the proposed IGNN model outperforms other comparable models. For example, the proposed IGNN model has presented the highest index values given the parameter R@20. Fig 7 compares the training results of each model on the Yelp dataset.

**Fig 8:**
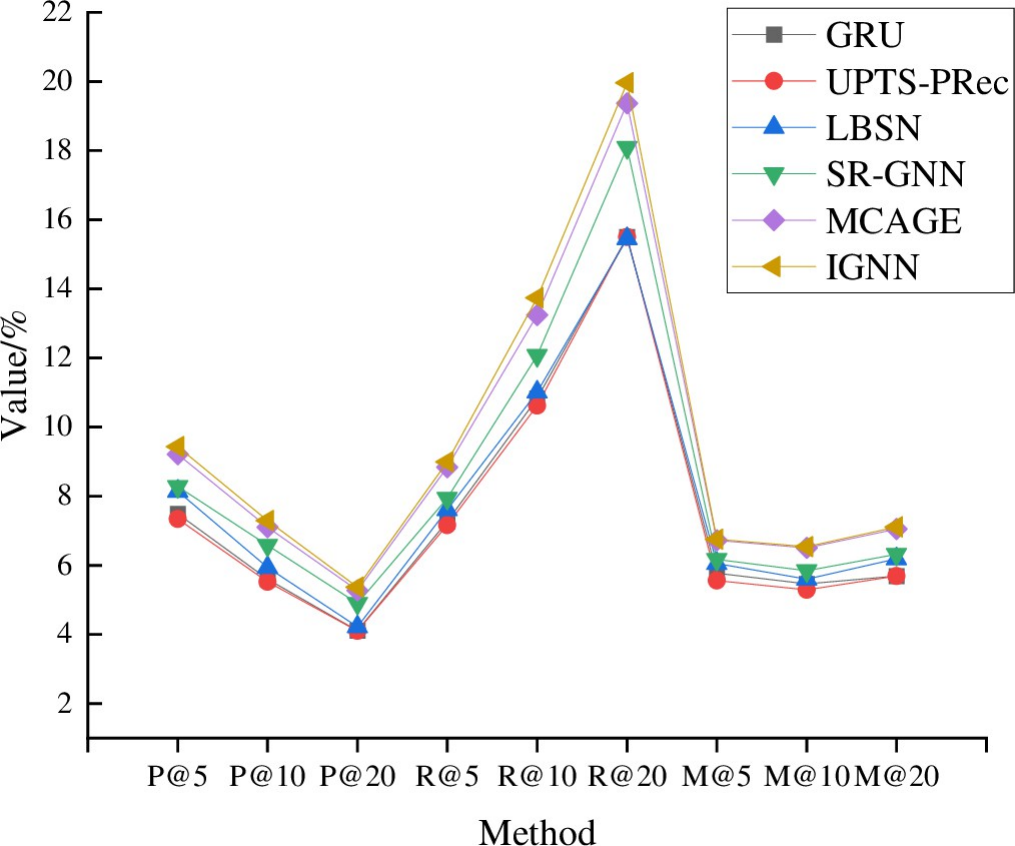
Model training results on the Foursquare dataset.

As in Fig 7, the proposed IGNN model still shows an excellent training effect on the Yelp dataset, beating other models on Precision, Recall, and Map indexes. When the model parameter is R@20, the proposed IGNN model gets the highest training index. Similarly, Fig 8 compares the training results of each model on the Foursquare dataset.

From Fig 8, the training results of the proposed IGNN model are still the best among all models. Nevertheless, compared with other datasets, the advantage of the proposed IGNN model on the Foursquare dataset is not that obvious. The highest index of the proposed IGNN occurs under the parameter P@20.

## Discussion

With the rapid development of the Internet and the popularity of mobile terminals, human civilization has entered the era of the Internet of Everything (IoE). Inevitably, marketing modes change radically, from product-centered to customer orientation to meet the spiritual needs of customers, shaping a new digital and intelligent marketing mode. Over time, the Immersive Marketing model emerged as a very advanced marketing model that provides important support for promoting commodity marketing. In this paper, a PRS is designed based on the DL + Immersive Marketing model. The proposed model can recommend user-interested commodities, enhance the user shopping experience, and promote sales. The proposed IGNN model presents an excellent performance by training different datasets and providing users with a pleasant shopping platform. The results show that Immersive Marketing has become the main marketing method in the business and entertainment sectors. Meanwhile, strengthening the marketing strength using the DL model can promote the future development of Immersive Marketing. On the Book-Crossings dataset, when the parameters are 5, 10, and 20, the IGNN model outperforms other models considerably. For example, when the model parameter is R@20, the proposed IGNN model gets the highest training index results. The IGNN model still shows excellent advantages in Precision, Map, and Recall on the Yelp dataset, with the highest values among all models. The peak training index is obtained under a parameter value R@20. Lastly, on the Foursquare dataset, the IGNN model performs excellently as well, only with a less obvious advantage than on the other two datasets. The optimal model index occurs at P@20. Compared with Hui et al.’s (2022) research, this paper provides a more advanced GNN model and PRS integrating Immersive Marketing. The proposed IGNN model can provide a shopping platform more in line with users’ psychology, greatly promote sales, and help entrepreneurs promote entrepreneurial activities [34].

## Conclusion

This paper integrates the DL NN model in the Immersive Marketing environment to design the DL NN-based PRS. The research framework is mainly based on the GNN. Then, the proposed model is used to predict users’ POI and give recommendations. The experimental findings corroborate that Immersive Marketing has become the primary commodity marketing mode. It can fully reflect commodities’ characteristics and fundamental attributes, comprehensively improve User Experience, and promote sales and the marketing environment. The IGNN model reported here outperforms other recommendation models in all index tests, and the optimal model parameters are P@20. Lastly, there are some defects in the present research. Model verification in practical application is absent. It fails to provide the impact of the proposed model on users. Therefore, this research will be strengthened in future research.

## Supporting information

S1 Data (RAR)
