# Correction: Exploration of consumer preference based on deep learning neural network model in the immersive marketing environment

**DOI:** 10.1371/journal.pone.0306470

**Published:** 2024-06-26

**Authors:** Qiang Zheng, Qing Shan Ding

The second author’s name is spelled incorrectly. The correct name is: Qing Shan Ding. The correct citation is: Zheng Q, Ding QS (2022) Exploration of consumer preference based on deep learning neural network model in the immersive marketing environment. PLOS ONE 17(5): e0268007. https://doi.org/10.1371/journal.pone.0268007.
